# Socioeconomic disparities in dietary diversity among adults in Bengbu, China: unraveling the mediating role of nutrition literacy

**DOI:** 10.3389/fnut.2025.1659550

**Published:** 2025-11-04

**Authors:** Yuhui Sun, Xiaoting Hu, Xi Tian, Huaqing Liu

**Affiliations:** School of Public Health, Bengbu Medical University, Bengbu, Anhui, China

**Keywords:** dietary diversity, nutrition literacy, socioeconomic status, adults, China

## Abstract

**Background:**

Socioeconomic status (SES) was linked to dietary diversity and nutrition literacy (NL) was associated with food choices. This study aims to investigate the association of NL with SES and dietary diversity, and to assess its potential mediating role between SES and dietary diversity among Chinese adults.

**Methods:**

A cross-sectional survey was previously conducted in Bengbu, China, including 2,208 adults aged ≥18. SES was primarily measured according to education level and monthly income. Dietary diversity scores (DDS) were collected through a simplified diet frequency questionnaire. NL was evaluated through a twelve-item short-form NL scale. Multiple linear regression and Hayes’ PROCESS framework were used for analysis.

**Results:**

Significant education- and monthly income-based disparities in dietary diversity were observed (both *p* < 0.001). After adjusting for confounding factors, each one-unit increase in education and monthly income was associated with a significant 0.29-point (95% confidence interval (CI): 0.20–0.37) and 0.22-point (95% CI: 0.13–0.30) increase in DDS, respectively, and with a 3.25-point (95% CI: 2.79–3.71) and 1.44-point (95% CI: 0.97–1.92) increase in NL, respectively. NL significantly mediated the association between monthly income or education and DDS (*β* = 0.180, 95% CI: 0.146–0.216; *β* = 0.136, 95% CI: 0.107–0.168, respectively).

**Conclusion:**

Sufficient NL can increase dietary diversity and plays a pivotal mediating role in the association between SES and dietary diversity. Intervention strategies should target populations with low SES and inadequate NL to improve dietary diversity and reduce economic inequalities in health.

## 1 Introduction

Dietary diversity refers to the variety of distinct food items or categories consumed by an individual over a specific period (1). It is widely recognized as a critical indicator within assessment systems for dietary nutrition levels. Appropriate dietary diversity can improve micronutrient intake and contribute to better nutritional status (2). Individuals with diverse diets tend to have higher intake of fiber, antioxidants, and other beneficial nutrients and consume fewer ultra-processed foods (3). Conversely, limited dietary diversity has been associated with a higher risk of obesity and chronic diseases, including cardiovascular diseases, diabetes, and metabolic syndrome (3–5). Furthermore, insufficient dietary diversity can cause micronutrient deficiencies that affect immune function and overall health, such as elevated likelihood of severe illnesses due to suboptimal vitamin D and zinc intake (6). Thus, the promotion of dietary diversity is a vital strategy in chronic disease prevention and management. Appropriate variety in food consumption helps ensure the intake of a diverse range of nutrients, improving an individual’s nutritional status and diminishing health risks.

Socioeconomic status (SES), a key determinant of dietary diversity (7), encompasses economic, social, and work status, which are typically assessed based on education level, income, and occupation (8). These factors collectively influence an individual’s ability to acquire resources and therefore also their dietary choices. Individuals in higher-status occupational strata tend to have a higher absolute expenditure on groceries, and this increased spending is associated with improved dietary quality (9). Consequently, individuals in medium to high SES brackets generally exhibit better health profiles and higher dietary quality than those in lower SES brackets (10, 11). In contrast, individuals with lower SES often must purchase low-nutrient, poor-quality food products due to their cost-effectiveness (12). This economic disparity creates differential access to nutritionally dense foods—while higher-SES individuals enjoy better access to fruits, vegetables, and dairy products (13), lower-SES populations face systematic barriers to obtaining such foods. Although numerous studies have extensively documented the socioeconomic disparities in dietary diversity between populations, the factors underlying these disparities remain incompletely understood.

Nutrition literacy (NL), a burgeoning field of research derived from health literacy, refers to the capacity to access, analyze, and interpret nutritional information, which enables individuals to make informed food choices (14, 15). NL is a crucial determinant of dietary behaviors and consumption patterns (16). Several studies have indicated that individuals with high NL are more likely to select nutrient-rich foods, reduce intake of unhealthy options, and consequently improve their overall dietary quality (17, 18). Additionally, individuals with higher NL typically exhibit better adherence to national dietary guidelines and a greater capacity for nutritional self-management, whereas those with lower NL exhibit a diminished capacity to make informed food choices (16–19). Research further reveals that individuals with high NL are more likely to adhere to either the Mediterranean diet or a prudent dietary pattern characterized by increased consumption of vegetables and whole grains and decreased intake of processed foods (16). Moreover, individuals with high NL exhibit superior skill in interpreting food labeling information to those with low NL and can effectively avoid high-sugar and high-sodium products (20).

This study examined the mediating role of NL in socioeconomic disparities regarding dietary diversity among Chinese adults using data from a previously conducted cross-sectional survey. The results provide a theoretical framework and practical directives for tailored interventions to address socioeconomic disparities in nutritional health.

## 2 Materials and methods

### 2.1 Study design and participants

A cross-sectional survey on the association between NL and health was distributed from May to July 2023 among adults in Bengbu, China. Participants were selected through urban–rural stratified multistage random sampling (21). The sampling proceeded as follows: (1) random selection of four primary areas (two urban, two rural); (2) random selection of two sub-areas from each primary area; and (3) random selection of 110 households from each sub-area. All eligible members in these households were included in the study. Inclusion criteria for the survey’s participants were being older than 18 years, the average age was 60.8 years with the standard deviation of 16.3. Further details on the study design are presented in a previous work (21). Data were collected through a self-designed structured questionnaire. All participants were informed that their participation was voluntary, and subsequently provided signed informed consent. The study was approved by the Ethics Committee of Bengbu Medical College.

A total of 2,279 respondents completed the survey, among whom 71 (3.1%) were excluded due to missing data on education level, monthly income, and dietary diversity score (DDS) parameters. In the final analysis, 2,208 eligible responses were retained. The characteristics of the lost sample did not differ significantly from those of the final sample.

### 2.2 SES measurement

Education, income, and occupational status are typical SES indicators. Given the inherent inconsistency in occupational taxonomies and their context-dependent variability (22, 23), only education and income metrics were used to measure SES. Educational levels were classified into ‘primary school or below,’ ‘junior high school,’ and ‘high school or above,’ in accordance with the International Standard Classification of Education (ISCED). Monthly income was categorized into three levels: <1,000 RMB, 1,000–3,000 RMB, and ≥3,000 RMB.

### 2.3 Nutrition literacy assessment

The nutrition literacy-short form (NL-SF), adapted from a comprehensive 43-item NL assessment tool (24), was administered to evaluate NL and exhibited sound validity (25). The scale comprised six dimensions: knowledge, understanding, obtaining skills, applying skills, interactive skills, and critical skills. Participants rated each item on a 5-point scale. Item scores were aggregated, with higher scores reflecting greater NL.

### 2.4 Dietary diversity measurement

Dietary information was gathered through a simplified questionnaire that instructed participants to report their intake frequencies of meat, fish, eggs, beans, vegetables, fruits, milk, and nuts. The intake frequency of each food group was categorized as “almost every day,” “at least once a week,” “at least once a month,” “not every month, but occasionally,” and “rarely or never.” A score of 1 point was assigned if a food group was consumed “almost every day” or “at least once a week”; a score of 0.5 points was allocated for consumption “at least once a month”; otherwise, no points were allocated. The Dietary diversity scores (DDS) was calculated as the cumulative point total (0 to 8) across all eight food groups. Higher scores signified greater dietary diversity. Cereals and oil were excluded from the DDS because nearly all adults in China consume these foods daily (26, 27). The eight food groups selected conform to the categories outlined in the Chinese Food Pagoda (28), effectively representing dietary quality and diversity among Chinese adults. The scale demonstrated acceptable reliability with a Cronbach’s alpha coefficient of 0.74. The DDS has been widely recognized as an indicator of diversity in food categories, sources, and nutrients consumed by individuals and groups over a specified period (29), and it has been adopted globally as a key tool within nutritional assessment frameworks due to its operational simplicity and efficiency (30).

### 2.5 Covariates

Information on the following sociodemographic characteristics was collected: gender (female, male), age (18 to 65 years, 66 years or older), marital status (married, other), place of residence (urban, rural), occupational status (employee, retired staff, farmer, other), alcohol consumption (never, formerly, currently), smoking history (never, formerly, currently), tea consumption (yes, no), participation in physical exercise (never, formerly, currently), and body mass index (BMI; underweight, <18.5 kg/m^2^; normal, 18.5–23.9 kg/m^2^; overweight, 24–27.9 kg/m^2^; obese, ≥28 kg/m^2^) (31).

### 2.6 Statistical analysis

Statistical analyses were conducted with SPSS. Continuous variables are presented as means ± standard and categorical variables as frequencies and percentages. Continuous variables were compared through student’s t-test and analysis of variance. The associations between SES and both dietary diversity and NL were evaluated through multiple linear regression analysis. Controlling for potential confounding effects of covariates, multiple linear regression was utilized to examine the independent associations between socioeconomic status and the outcome variables (dietary diversity score and nutrition literacy) by calculating regression coefficients (*β*) and its 95% confidence interval (CI), which represent the average change in the outcome variable per one-unit increase in the predictor variables. The PROCESS mediation framework, developed by Hayes, was employed to analyze the pathways through which NL mediated the association between SES and dietary diversity. A *p* value < 0.05 was considered statistically significant.

## 3 Results

### 3.1 Basic characteristics

Table 1 presents the baseline characteristics of the study samples (*n* = 2,208). Among respondents, 48.9% were aged 18–65 years, 61.3% were female, 53.6% lived in rural areas, 77.9% were married, 48.2% had received a primary school or lower education, and 40.2% had a monthly income of less than 1,000 RMB. Participants who were younger, resided in urban areas, were employed, participated in exercise, and consumed tea exhibited relatively higher DDS.

**Table 1:** Participants’ demographic characteristics and dietary diversity.

Variable	*N*	DDS (Mean ± SD)	*t*/*F*	*p*
Total	2,208	6.20 (1.44)		
Age (year)			8.84	<0.001
18–65	1,080 (48.9)	6.50 (1.29)		
66 and above	1,068 (48.4)	5.96 (1.49)		
Gender			1.19	0.243
Male	857 (38.7)	6.25 (1.37)		
Female	1,351 (61.3)	6.17 (1.48)		
Place of residence			13.84	<0.001
Urban	1,024 (46.4)	6.63 (1.24)		
Rural areas	1,184 (53.6)	5.83 (1.49)		
Marital status			0.84	0.404
Married	1721 (77.9)	6.22 (1.45)		
Others	487 (22.1)	6.16 (1.39)		
Education level			237.71	<0.001
Primary school and below	1,065 (48.2)	5.61 (1.45)		
Junior high school	479 (21.7)	6.46 (1.26)		
High school and above	664 (30.1)	6.98 (1.06)		
Type of occupation			53.42	<0.001
Employees	227 (10.3)	6.87 (1.14)		
Separated/retired staff	722 (32.8)	6.45 (1.38)		
Farmers	687 (31.2)	5.72 (1.42)		
Others	566 (25.7)	6.21 (1.45)		
Monthly income (¥)			175.56	<0.001
<1,000	887 (40.2)	5.59 (1.51)		
1,000–3,000	679 (30.8)	6.40 (1.29)		
3,000 and above	642 (29.1)	6.84 (1.09)		
Smoking			0.38	0.686
No	1,602 (72.6)	6.22 (1.46)		
Former smoker	199 (9.0)	6.21 (1.46)		
Current smoker	407 (18.4)	6.15 (1.34)		
Alcohol drinking			6.33	0.002
No	1,408 (63.8)	6.13 (1.49)		
Former drinker	254 (11.5)	6.25 (1.38)		
Current drinker	546 (24.7)	6.38 (1.29)		
Physical exercise			52.29	<0.001
Exercised never	659 (29.9)	5.73 (1.47)		
Exercised previously	249 (11.3)	6.41 (1.47)		
Exercise currently	1,296 (58.8)	6.40 (1.36)		
Drink tea			5.23	<0.001
Yes	755 (34.5)	6.42 (1.37)		
No	1,433 (65.5)	6.09 (1.50)		
BMI			3.65	0.012
<18.5 kg/m^2^	58 (2.7)	5.89 (1.44)		
18.5–23.9 kg/m^2^	874 (40.4)	6.31 (1.45)		
24–27.9 kg/m^2^	856 (39.6)	6.19 (1.41)		
≥28 kg/m^2^	374 (17.3)	6.07 (1.41)		

SD, standard deviation; *t*/*F*, respectively represent the result of *t*-test and analysis of variance (ANOVA).

### 3.2 Associations between SES, DDS, and NL

Table 2 presents NL scores by socioeconomic status, showing that respondents with higher education levels and greater monthly income had significantly higher NL scores (*p* < 0.05). As indicated in Table 3, after adjusting for potential confounders (age, gender, place of residence, marital status, education, occupation, monthly income, smoking, alcohol consumption, current exercise status, tea drinking, and BMI), both components of SES showed significant positive associations with DDS and nutrition literacy. Specifically, higher education level was associated with greater DDS and higher nutrition literacy. Similarly, higher monthly income was linked to increased DDS and improved nutrition literacy. Additionally, nutrition literacy itself was positively associated with DDS.

**Table 2:** Score of nutrition literacy according to socioeconomic status.

Variable	*N*	Nutrition literacy (Mean ± SD)	*F*	*p*
Education level			441.95	<0.001
Primary school and below	1,065 (48.2)	33.21 (7.23)		
Junior high school	479 (21.7)	39.31 (7.60)		
High school and above	664 (30.1)	43.64 (6.94)		
Monthly income (¥)			241.42	<0.001
<1,000	887 (40.3)	33.52 (7.51)		
1,000–3,000	679 (30.8)	38.87 (8.27)		
3,000 and above	642 (29.1)	42.12 (7.48)		

SD, standard deviation.

**Table 3:** Association between socioeconomic status, dietary diversity and nutrition literacy.

Variable	DDS *β* (95%CI)	*p*	Nutrition literacy *β* (95%CI)	*p*
Education level	0.29 (0.20, 0.37)	<0.001	3.25 (2.79, 3.71)	<0.001
Monthly income	0.22 (0.13, 0.30)	<0.001	1.44 (0.97, 1.92)	<0.001
Nutrition literacy	0.05 (0.04, 0.05)	<0.001		

95% CI represents 95% confidence intervals. Multiple linear regression analysis was adjusted for age, gender, place of residence, marital status, education, occupation, monthly income, smoking, alcohol consumption, current exercise status, drinking tea and BMI.

### 3.3 Mediating effect of NL on the association between SES and dietary diversity

**Figure 1:**
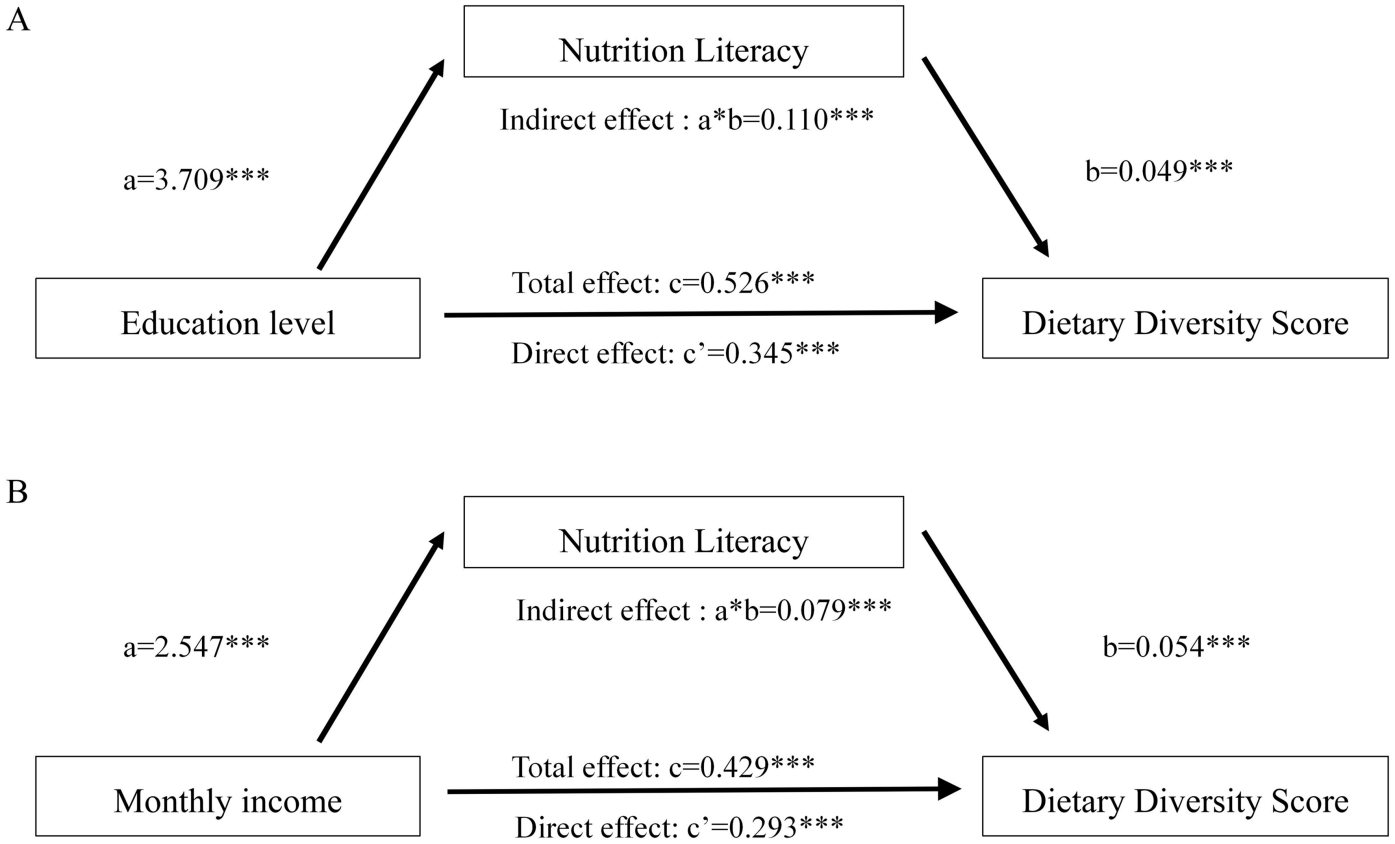
The mediation model between socioeconomic status, nutrition literacy, and dietary diversity score after adjusting confounders. **(A)** Mediating effect of nutrition literacy on the association between education and dietary diversity. **(B)** Mediating effect of nutrition literacy on the association between monthly income and dietary diversity. c: the total effect of independent variable X on dependent variable Y. a: the effect of independent variable X on mediator M. b: the effect of mediator M on dependent variable Y after controlling for independent variable X. c’: the direct effect of independent variable X on dependent variable Y after controlling for mediator M. *p < 0.05; **p < 0.01; ***p < 0.01.

Figure 1 illustrates the mediating role of nutrition literacy in the relationship between SES and dietary diversity. For education level, the direct effect on dietary diversity was 0.345 (*p* < 0.001), while the indirect effect mediated through NL was 0.110 (*p* < 0.001), accounting for 20.9% of the total effect (0.526). Similarly, for monthly income, the direct effect on dietary diversity was 0.293 (*p* < 0.001), with an indirect effect via NL of 0.079 (*p* < 0.001), representing 18.4% of the total effect (0.429). In both cases, the confidence intervals for the indirect effects did not include zero, confirming the significant mediating role of NL.

## 4 Discussion

This study is the first to evaluate NL as a mediator in the association between SES and dietary diversity among adults. Higher SES was significantly associated with greater dietary diversity and NL. Furthermore, NL mediated the association between SES and dietary diversity. These findings suggest the need for synergistic intervention strategies targeting both NL and SES to improve dietary diversity in adults.

Education level and monthly income were significantly associated with dietary diversity. Alkerwi (32) reported a socioeconomic disparity in dietary quality, in which educational level was significantly correlated with the ability to make adequate, healthy food choices and economic resources were the primary factor associated with dietary diversity. Higher education allows individuals to better comprehend the necessity of a healthy diet and make dietary choices informed by nutritional guidelines, promoting dietary diversity (33). Individuals with higher incomes generally possess greater purchasing power, granting them easier access to nutrient-dense and varied foods (e.g., fresh fruits, vegetables, whole grains, and low-fat dairy products). By contrast, socioeconomically disadvantaged populations typically rely on energy-dense but nutritionally inferior food sources due to financial constraints (34). Consequently, it is necessary to strengthen the attention to groups with low education levels and low incomes in order to improve their dietary diversity and enhance the quality of their diets.

NL emerged as a crucial mediator of the association between SES and dietary diversity. Individuals with high NL are capable of critically inspecting nutrition labels (20) and exhibit a preference for foods containing key nutrients (35, 36)—a tendency rooted in their strong general awareness of the nutritional value of different food groups (14). This awareness, derived from their better understanding of nutritional profiles across food categories, motivates them to make diversified food choices. Additionally, individuals with high-NL are able to transfer their nutritional knowledge into practice by using lower-fat cooking techniques, making better food combinations (37, 38), and ultimately increasing their dietary diversity (39).

Individuals with high SES typically exhibit higher NL (15) and are more likely to form healthy dietary habits than those with low SES. Educational level, a key driver of NL, enables high-education individuals to better obtain and interpret complex nutritional information (e.g., nutrition fact labels, dietary guidelines) and to proactively seek authoritative scientific information (40–42)—skills that help them align their dietary patterns with guidelines. High-income individuals further benefit from diverse knowledge-acquisition channels such as professional lectures, scientific publications and customized online courses (43), which enhance their ability to apply nutritional knowledge to daily diets. In contrast, low-income individuals often rely on general media or nonprofessional sources for nutritional information (44), contributing to dietary diversity disparities across SES brackets. However, high SES does not necessarily correspond to a highly diverse diet. One study reported that although participants with high education levels had adequate knowledge about healthy eating behaviors, their dietary intake still included high-sugar and high-sodium foods (45), which might be due to time scarcity, preference for convenience, or underestimation of long-term health risks. Additionally, some individuals with high-SES may prefer expensive processed foods with poor nutritional quality due to low NL (46). These findings indicate that high SES can boost dietary diversity only among those with high NL. Therefore, NL is a vital element of interventions designed to improve dietary diversity and to promote healthy diets.

This study has several limitations. First, the DDS evaluation was based on self-reported dietary data collected with a food frequency questionnaire covering the 12-month period prior to administration and may have been subject to recall bias. Second, the respondents were recruited from Bengbu, China, and their dietary patterns may not fully represent those of populations in different geographical regions, potentially limiting the generalizability of the findings. Third, this study lacked detailed data on household composition (e.g., co-residence with children or elderly parents). Participants who cook for family members may have their dietary choices influenced by dependents’ preferences and nutritional needs. Future research should include household structure as a potential confounding or moderating factor when analyzing dietary diversity. Finally, the cross-sectional study design limited the ability to establish causal relationships between variables. Future longitudinal studies should expand the sample coverage to establish causality and further validate the proposed hypotheses.

## 5 Conclusion

Dietary diversity is influenced by an individual’s socioeconomic status. Sufficient NL can increase dietary diversity and is a key mediator of the association between SES and dietary diversity. Intervention strategies should target populations with low SES and inadequate NL to improve DDS and reduce economic inequalities in health. Intervention priorities can include nutrition-related education campaigns and intervention programs to simultaneously improve NL and dietary diversity in adults, which can improve population-level health and nutritional equity, in alignment with sustainable development goals.

## Data Availability

The datasets presented in this article are not readily available because the data supporting the findings of this study are available from the corresponding author upon reasonable request for collaboration. Requests to access the datasets should be directed to Huaqing Liu, hqliu@bbmu.edu.cn.
